# Involved-field radiotherapy in limited-stage small-cell lung cancer

**DOI:** 10.1038/sj.bjc.6603862

**Published:** 2007-06-26

**Authors:** C-R Chien, C-J Yu

**Affiliations:** 1 Department of Oncology, National Taiwan University Hospital and College of Medicine, National Taiwan University, Taipei, Taiwan, Republic of China; 2 Department of Internal Medicine, National Taiwan University Hospital and College of Medicine, National Taiwan University, Taipei, Taiwan, Republic of China

**Sir**,

Elective nodal irradiation (ENI) was used in conventional radiotherapy (RT) target volume for limited-stage small-cell lung cancer (LS-SCLC) in contrast to involved-field RT only in some recent studies (Erridge and Murray, 2003). In contrast to non-small-cell lung cancer (NSCLC), where ENI was now less favoured (Senan *et al*, 2004), whether ENI could be safely omitted or not was less clear for LS-SCLC. To our knowledge, your article by Baas *et al* (2006) was the first prospective report regarding involved-field RT (i.e., omission of ENI). However, we felt that the data and conclusion reported in this study were not complete. The authors reported that local recurrences (within the target area) were seen in six patients (16%). It was concluded that combination chemotherapy with concurrent involved-field RT is an effective treatment for LS-SCLC. However, the number of nodal failures (within omitted ENI) was not reported. De Ruysscher *et al* (2006) reported that three patients (crude rate 11%, 95% CI 2.4–29%) developed an isolated nodal failure in a phase II study employing ENI. They concluded that the safety of selective nodal irradiation in NSCLC should not be extrapolated to patients with LS-SCLC until more data are available. We believe that the detail recurrence pattern in the study by Baas *et al* (2006) would be of great value in the issue of ENI in RT for LS-SCLC if this was reported formally in your journal, unless it was planned to be published elsewhere.
